# Antidepressant-Like Effects of *n*-Butylidenephthalide Using In Vivo and In Silico Approaches

**DOI:** 10.3390/ph19020242

**Published:** 2026-01-30

**Authors:** María Leonor González-Rivera, María del Carmen Juárez-Vázquez, Athzirys Alejandra Melecio-Hernández, Diana Casique-Aguirre, Gabriela Josefina López-González, Ramsés Maximiliano Ramírez-Martínez, Andrea Ayala-Torres, Yurisleidys Quesada-Mendiola, Juan Ramón Zapata-Morales, Angel Josabad Alonso-Castro

**Affiliations:** 1 Department of Pharmacy, Natural and Exact Sciences Division, University of Guanajuato (UG), Guanajuato 36200, Mexico; carmen.juarez@ugto.mx (M.d.C.J.-V.); aa.meleciohernandez@ugto.mx (A.A.M.-H.); d.casiquea@ugto.mx (D.C.-A.); gj.lopezgonzalez@ugto.mx (G.J.L.-G.); rm.ramirez.martinez@ugto.mx (R.M.R.-M.); a.ayalatorres@ugto.mx (A.A.-T.); y.quesadamendiola@ugto.mx (Y.Q.-M.); juan.zapata@ugto.mx (J.R.Z.-M.); 2 Secretariat of Science Humanities Technology and Innovation, Mexico City 03940, Mexico

**Keywords:** antidepressant, motor coordination, isobenzofuranone, anxiolytic

## Abstract

**Background:** The plant-derived compound n-butylidenephthalide (BP) is an isobenzofuranone that has shown neuropharmacological effects on preclinical models of Parkinson’s, Alzheimer’s, and amyotrophic lateral sclerosis. **Methods**: This study evaluated the anti-inflammatory, antinociceptive, anxiolytic-like, hypnotic, anticonvulsant, and antidepressant-like effects of BP (50–200 mg/kg p.o.) using Balb/c mice in acute assays. This study also evaluated the antidepressant-like effects of BP in a mouse model of reserpine-induced depression-like behavior for 20 days. Inhibitors involved in the molecular process of depression and in silico studies were used to evaluate a possible mechanism of action for the antidepressant-like effects of BP. **Results**: BP induced low anti-inflammatory effects, showed low anticonvulsant effects, and lacked hypnotic effects or motor impairment in acute assays. The antidepressant-like effects of BP (100–200 mg/kg p.o.) were comparable to amitriptyline (25 mg/kg p.o.) in acute assays. The participation of serotonergic and adrenergic systems is involved in the acute antidepressant-like effects of BP. In the reserpine-induced depression model, BP (100 mg/kg p.o.) showed antidepressant-like effects in one of the two antidepressant tests, but with a lower effect than amitriptyline (20 mg/kg p.o.). **Conclusions:** BP (100 and 200 mg/kg) showed antidepressant-like effects in acute assays and, to a lesser extent, in a reserpine-induced chronic model of depression-like behavior.

## 1. Introduction

Persistent depressive disorder (PDD) is characterized by the avoidance of social activity, sleep problems, loss of interest in daily activities, poor concentration, and trouble in making decisions for long periods of time. Approximately 4% of the world’s population and 5.7% of adults (70 years or older) can suffer from PDD [1]. Women are more susceptible to experiencing PDD, and alcohol and physical inactivity are the main factors that influence PDD [1]. Antidepressant medications such as fluoxetine and amitriptyline (AMT) are commonly used for the treatment of PDD. However, these medications induce adverse reactions such as weight gain, dizziness, constipation, and headache, among others [2]. Furthermore, inflammation is involved in the progression of mental disorders [3]. Patients with major depressive disorder (MDD) exhibit an increase in the serum levels of inflammatory molecules such as tumor necrosis factor (TNF-α) and interleukin (IL)-1β [4]. In the context of the need for the development of new treatments, medicinal plants have demonstrated clinical efficacy with activity comparable to that of reference drugs in the treatment of mental disorders [5]. Consequently, medicinal plants and their active compounds may serve as potential therapies for mental disorders [3].

The compound n-butylidenephthalide (BP) was first isolated and identified in the chloroform extract from *Angelica sinensis* (Oliv.) Diels (Apiaceae), a medicinal plant used in traditional Chinese medicine for the treatment of menopausal symptoms, hypertension, inflammation, pain, and spasms [6]. The effects of BP on in vitro and in vivo models of neurological diseases have been tested, including Parkinson’s, Alzheimer’s, and amyotrophic lateral sclerosis [7,8,9]. BP (2 and 5 mM) was administered for 72 h in a transgenic *C. elegans* model that expresses human α-synuclein in muscle cells. This treatment with BP reduced dopaminergic neuron degeneration and decreased alpha-synuclein accumulation [7]. These findings suggest neuroprotective effects in a Parkinsonism-like model. Furthermore, BP (50 and 100 mg/kg p.o.) was administered daily for 13 weeks, which led to a reduction in motor impairment in a model of spinocerebellar ataxia type 3 using C57BL/6 transgenic mice that expressed the human ATXN3, a marker of toxicity in neurons. BP also decreased the toxic fragment formation of ATXN3 [10]. Another study showed that BP (120 mg/kg p.o.) improved short-term memory, with comparable activity to 10 mg/kg donepezil, and decreased amyloid accumulation in the hippocampus and cortex of 3xTg-AD 9-month-old transgenic mice, which exhibited synaptic dysfunction, neuropathy, plaques, and neurofibrillary tangles [8]. That study suggested a possible effect of BP on Alzheimer’s disease. Finally, BP (10 and 20 µM) after 72 h of treatment prevented degeneration and apoptosis and decreased glutamate-induced calcium influx in neurons using a human-induced pluripotent stem cell-based amyotrophic lateral sclerosis model [9].

There is limited information on the pharmacokinetic profile of BP. An intravenous injection of BP revealed that this compound is metabolized to a polar form, a cysteine conjugate, and excreted into urine [11]. The metabolic profile of BP in rat and human liver microsomes revealed the presence of 14 metabolites. BP is metabolized by hydroxylation, hydration, hydrolysis, and glutathione conjugation [12].

Finally, an ethanol extract of the orchid *Stanhopea tigrina* Bateman ex Lindl. (Orchidaceae) leaves (10–100 mg/kg p.o.) showed acute anxiolytic-like actions in mouse models with the possible participation of GABAergic, adrenergic, and serotonergic systems. BP was one of the compounds identified in this ethanol extract, and its content represented 2.36% of the plant extract [13]. Other medicinal plants such as *Ligusticum jeholense* (Nakai & Kitag.) Nakai & Kitag. (Apiaceae) and *Pleurospermum angelicoides* (DC.) Benth. ex C.B. Clarke (Apiaceae) contained BP amounts of 18.7% and 6.5%, respectively [14].

This study aimed to evaluate the antidepressant-like, anxiolytic-like, hypnotic, anticonvulsant, anti-inflammatory, and antinociceptive effects of BP (50–200 mg/kg p.o.) using murine models in acute assays. This study also evaluated the antidepressant-like effects and the possible mechanism of action of BP in a mouse model of reserpine-induced depression-like behavior for 20 days.

## 2. Results

### 2.1. Acute Toxicity

**Figure 1:**
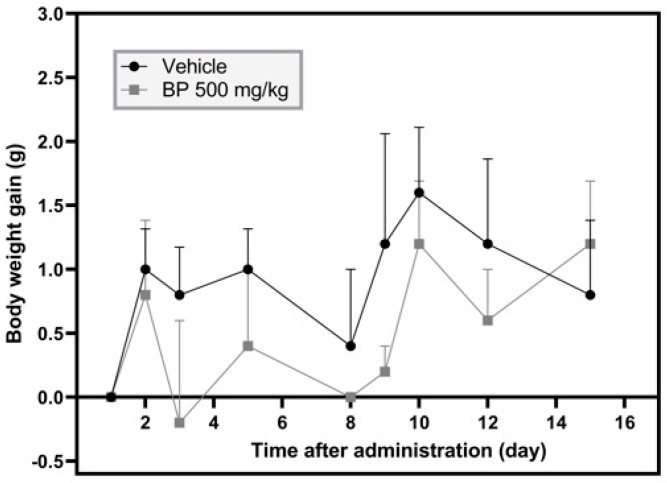
Body weight gain registered during the acute toxicity test performed with a single administration of BP (500 mg/kg p.o.) and the vehicle consisting of a mixture containing Tween 20 and water [1:9]. The results are reported with the mean ± standard error of the mean (SEM) (n = 8). Data were analyzed using a one-way analysis of variance (ANOVA) followed by Tukey post hoc tests.

Mice displayed no visible signs of toxicity (i.e., anorexia, unusual respiratory patterns, piloerection, immobility, etc.) following 15 days of single-dose administration of BP (500 mg/kg p.o.). The BP-treated mice exhibited no changes in body weight compared to the vehicle group (Figure 1). Weights of the liver, kidney, and spleen in both the vehicle and BP groups showed no differences, and no macroscopic abnormalities (changes in shape or color) were noted. Mice receiving BP (500 mg/kg p.o.) showed lethargy after 30 min post-treatment, but this effect was not shown after 1 h post-treatment.

### 2.2. Anti-Inflammatory Effects

**Figure 2:**
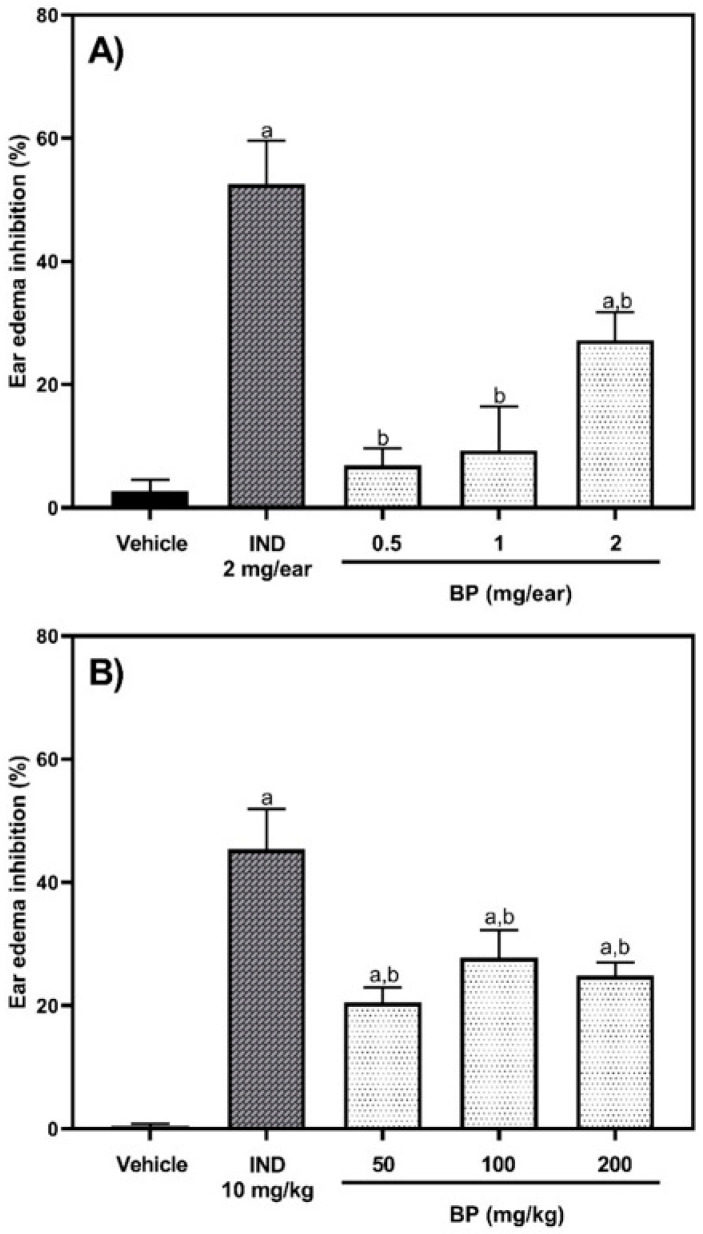
Effect of BP on 12-O-tetradecanoylphorbol-13-acetate (TPA)-induced ear edema testing administered topically (**A**) and orally (**B**). The abbreviations of IND and BP correspond to indomethacin (positive) and n-butylidenephthalide, respectively. The results are reported with the mean ± standard error of the mean (SEM) (n = 8). Data were analyzed using a one-way analysis of variance (ANOVA) followed by Tukey post hoc tests. The “a” letter means statistical difference (*p* < 0.05) versus the vehicle group, and “b” means statistical difference versus the IND group (*p* < 0.05).

The results of the 12-O-tetradecanoylphorbol-13-acetate (TPA)-induced ear edema test are illustrated in Figure 2. One-way ANOVA showed that there were significant differences between treatment groups administered topically [F (4,18) = 16.79, *p* < 0.0001] and orally [F (4,32) = 15.61, *p* < 0.0001)]. Only the topical application of 2 mg BP decreased (*p* < 0.05) ear edema by 27.2% compared to the vehicle group (*p* = 0.0190). However, the anti-inflammatory effect of BP (0.5, 1, and 2 mg/ear) was lower (*p* < 0.05) than the one observed by the reference drug (2 mg indomethacin), which reduced ear edema by 52.55% (Figure 2A). When BP was orally administered, there was a reduction in ear edema of 20.52% (*p* = 0.0120 versus the vehicle group), 27.75% (*p* = 0.0004 versus vehicle group), and 24.86% (*p* = 0.0036) at doses of 50, 100, and 200 mg/kg, respectively, compared to the vehicle group. These effects of BP were lower than those obtained with 10 mg/kg p.o. indomethacin (IND), which was 45.38% (*p* < 0.0001 versus vehicle group) (Figure 2B).

**Figure 3:**
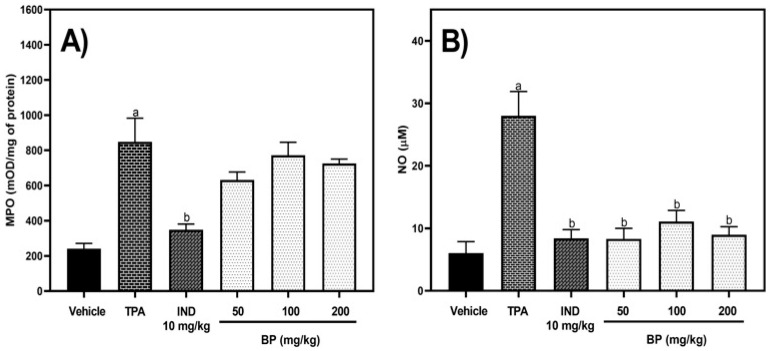
Effect of BP (50–200 mg/kg p.o.) on levels of MPO (**A**) and nitric oxide (NO) (**B**) in the 12-O-tetradecanoylphorbol-13-acetate (TPA)-induced ear edema test. The abbreviations of IND and BP correspond to indomethacin (positive) and butylidenephthalide, respectively. The results are reported with the mean ± standard error mean (SEM) (n = 6–8). Data were analyzed using a one-way analysis of variance (ANOVA) followed by Tukey post hoc tests. The vehicle group contemplates homogenates of the ear without TPA. The “a” letter means statistical difference (*p* < 0.05) versus the vehicle group, and “b” means statistical difference versus the TPA group (*p* < 0.05).

Additionally, the effect of BP administered orally on the levels of MPO and NO in the homogenates of ears was evaluated in the 12-O-tetradecanoylphorbol-13-acetate (TPA)-induced ear edema test. One-way ANOVA showed that there were significant differences between treatment groups in the levels of MPO [F (5,44) = 4.952, *p* = 0.0011] and NO [F (5,35) = 10.62, *p* < 0.0001]. Also, in the right-ear homogenates from the TPA group, the MPO levels increased 2.51-fold compared to the vehicle group (without TPA) (*p* = 0.0045). Only the ear homogenates from the indomethacin (10 mg/kg p.o.) group exhibited a reduction in MPO levels by 41.14% compared to the TPA group (*p* = 0.0094). In contrast, BP (50–200 mg/kg p.o.) showed no reduction in MPO levels compared to the TPA group (*p* > 0.05) (Figure 3A). On the other hand, the TPA group increased the nitric oxide levels 3.61 times compared to the vehicle group (without TPA) (*p* < 0.0001). The experimental groups treated with indomethacin (10 mg/kg p.o.) or with BP (50–200 mg/kg p.o.) showed reductions of 60.40% to 70.32% in nitric oxide levels compared to the TPA group (*p* = 0.0002 to 0.0015). No difference was shown between them in the ear homogenates (*p* > 0.05) (Figure 3B).

**Figure 4:**
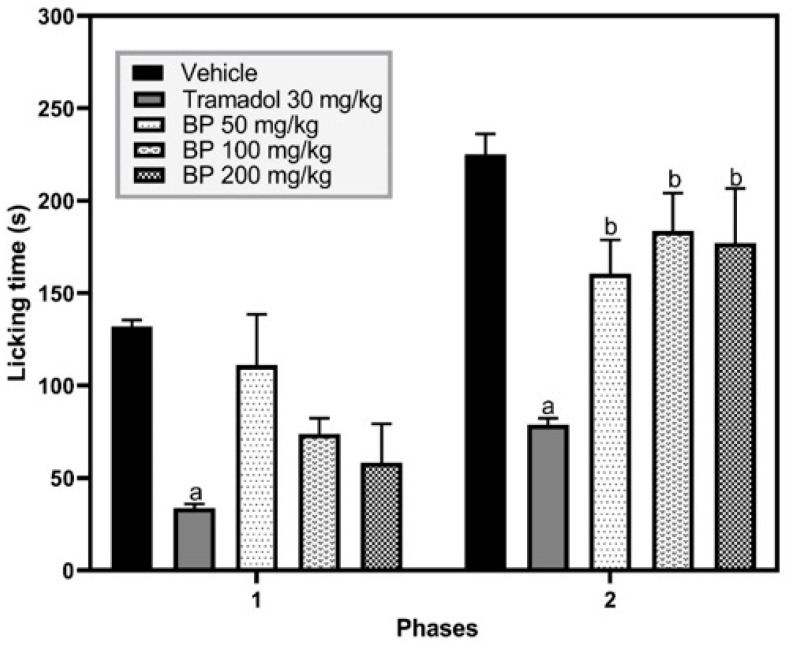
Effect of BP in the formalin model. The abbreviation BP corresponds to butylidenephthalide. The results are presented as the mean ± standard error of the mean (SEM) (n = 8). Data from each phase of the formalin test were analyzed using a one-way analysis of variance (ANOVA) followed by Tukey post hoc tests. The “a” letter means statistical difference (*p* < 0.01) versus the vehicle group, and “b” means statistical difference versus the tramadol group (*p* < 0.05).

In the formalin model, one-way ANOVA revealed significant differences between treatment groups in both the one-phase [F (4,31) = 4.213, *p* = 0.0077] and two-phase [F (4,27) = 7.544, *p* < 0.0003] assessments. The reference drug tramadol (30 mg/kg p.o.) decreased the licking time of the right hind paw in phase 1 (33.6 ± 2.4) and phase 2 (78.8 ± 3.5) when compared to the vehicle group, which had licking times of 127.7 ± 5.2 and 225.1 ± 11.15 in phases 1 and 2, respectively (*p* < 0.01). In contrast, BP (50–200 mg/kg p.o.) did not significantly reduce licking time in any phase of the formalin test (*p* > 0.05 versus vehicle group) (Figure 4).

### 2.3. Anxiolytic-Like Effects of BP

**Figure 5:**
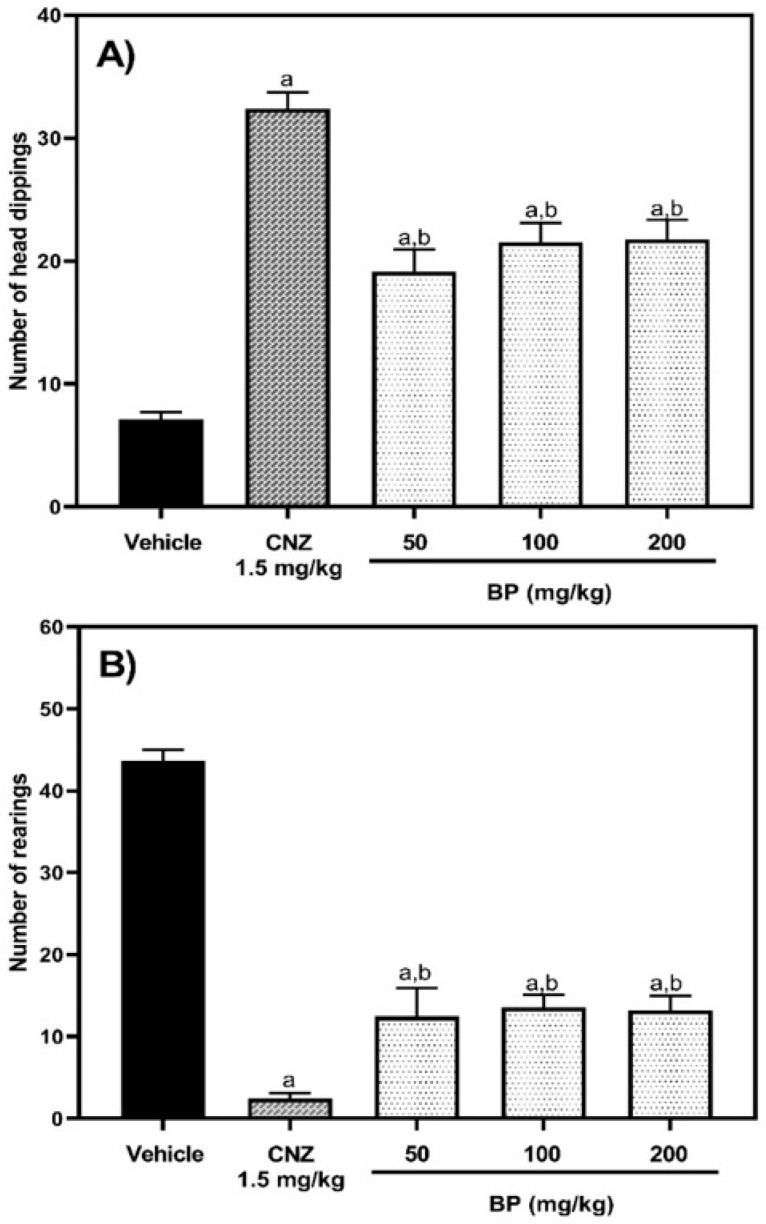
Anxiolytic-like activity of BP (50, 100, and 200 mg/kg p.o.) evaluated in HBT (**A**) and ECT (**B**). The reference drug was 1.5 mg/kg p.o. clonazepam (CNZ). The results of all experimental groups are reported as the mean values (±SEM) (n = 8). Data were analyzed using a one-way analysis of variance (ANOVA) followed by Tukey post hoc tests. In the HBT: ap < 0.0001 vs. the vehicle group, and bp = 0.0001 vs. the CNZ group. In the ECT: ^a^ *p* < 0.0001 vs. the vehicle group, and ^b^ *p* < 0.05 vs. the CNZ group.

In the hole-board test (HBT), one-way ANOVA analysis revealed significant differences between treatment groups [F (4,37) = 34.98, *p* < 0.0001]. Mice treated with 1.5 mg/kg clonazepam (CNZ) displayed a significantly (*p* < 0.0001) higher number of head dippings (3.5-fold) compared to the vehicle group. Mice treated with BP (50–200 mg/kg) also showed an increase in head dippings (1.7–2.0-fold) relative to the vehicle group, although this effect was dose-independent. However, the effects observed with BP were lower than those recorded by CNZ (1.5 mg/kg p.o.) (*p* = 0.0001) (Figure 5A).

In the exploratory cylinder test (ECT), one-way ANOVA analysis indicated significant differences between treatment groups [F (4,29) = 48.08, *p* < 0.0001]. All experimental groups exhibited a decrease in the number of rearings compared to the vehicle group (*p* < 0.0001). The reduction in rearings for the CNZ-treated group (1.5 mg/kg p.o.) was 94.4%, whereas the BP-treated groups (50–200 mg/kg p.o.) showed reductions in rearings ranging from 68.3 to 71.5% (*p* < 0.05 compared to the CNZ group only) (Figure 5B).

### 2.4. Sedative and Locomotor Effects of BP

**Figure 6:**
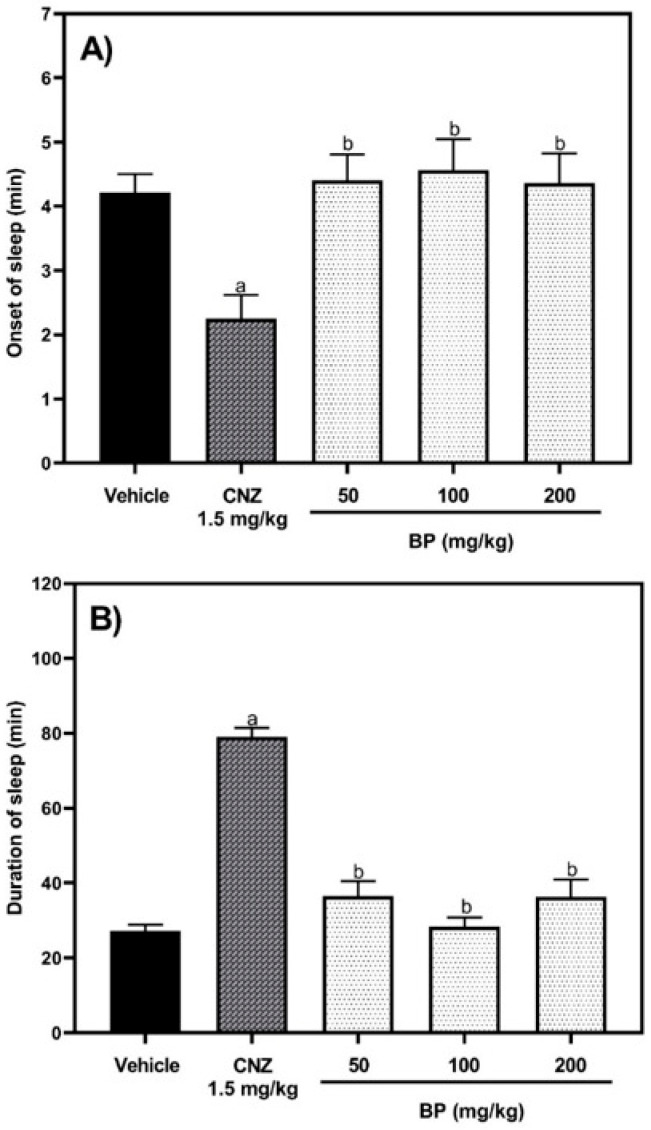
Sedative and hypnotic effects of BP. The hypnotic activity of BP (50, 100, and 200 mg/kg p.o.) was determined by the onset of sleep (**A**) and length of sleep (**B**) in the pentobarbital-induced sleeping test. Clonazepam (CNZ) (1.5 mg/kg p.o.) was the reference drug. The results of all experimental groups are reported as the mean values ± SEM (n = 8). Data were analyzed using a one-way analysis of variance (ANOVA) followed by Tukey post hoc tests. In (**A**), ap < 0.01 vs. the vehicle group, bp < 0.01 vs. the CNZ group. In (**B**) ^a^ *p* < 0.0001 vs. the vehicle group, ^b^ *p* < 0.0001 vs. the CNZ group.

In the pentobarbital-induced sleeping test, one-way ANOVA showed that there were significant differences between treatment groups in the onset time [F (4,38) = 5.634, *p* = 0.0012] and duration [F (4,35) = 44.26, *p* < 0.0001] of sedation. The mice treated with 1.5 mg/kg p.o. clonazepam (CNZ) showed a shorter onset time for sedation compared to the vehicle group (*p* = 0.0061). In contrast, the onset of sedation in the groups treated with BP (50–200 mg/kg) showed no statistically significant differences (*p* > 0.05) when compared to the vehicle group (Figure 6A). Also, the CNZ group presented a longer duration of sedation compared to the vehicle group and the groups treated with different doses of BP (*p* < 0.0001). Likewise, the BP-treated groups (50–200 mg/kg p.o.) exhibited the same duration of sedation as the vehicle group (*p* > 0.05) (Figure 6B).

**Figure 7:**
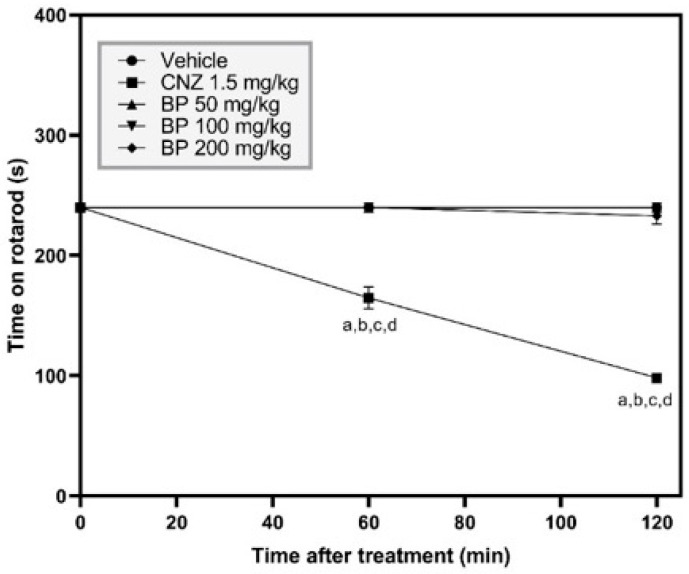
Locomotor effects of BP (50–200 mg/kg p.o.) and CNZ (1.5 mg/kg p.o.) on a rotarod device. The results of all experimental groups are reported as the mean values (±SEM) (n = 8). Data for each period (0, 60, and 120 min after administration of treatments) were analyzed using a one-way analysis of variance (ANOVA) followed by Tukey post hoc tests. ^a^ *p* < 0.05 vs. the vehicle group, ^b^ *p* < 0.05 vs. the BP 50 mg/kg group, ^c^ *p* < 0.05 vs. the BP 100 mg/kg group, and ^d^ *p* < 0.05 vs. the BP 200 mg/kg group.

In the rotarod test, one-way ANOVA revealed significant differences among the experimental groups at one hour [F (4,28) = 62.73, *p* < 0.0001] and two hours [F (4,28) = 284.9 *p* < 0.0001] following the administration of the treatments. BP (50–200 mg/kg p.o.) did not affect the motor coordination of the mice at any time while using the rotarod device. In contrast, CNZ (1.5 mg/kg p.o.) decreased the time spent on the rotarod by 31.3% (60 min) and 59% (120 min) (Figure 7).

### 2.5. Anticonvulsant Activity

In the pentylenetetrazol (PTZ)-induced convulsion test, one-way ANOVA revealed significant differences between experimental groups in both the onset [F (4,36) = 51.70, *p* < 0.0001] and the duration of convulsions [F (4,43) = 4.484, *p* = 0.0041]. CNZ (1.5 mg/kg p.o.) significantly (*p* < 0.0001) delayed the onset of convulsion and reduced (*p* = 0.0013) the duration of seizures when compared to the vehicle group. In contrast, BP (50–200 mg/kg p.o.) exhibited a similar (*p* > 0.05) pattern concerning both the onset and duration of convulsions relative to the vehicle group. CNZ (1.5 mg/kg p.o.) protected mice from mortality, and BP caused mortality in mice at 25% (BP 50 mg/kg) and 50% (BP at 100 and 200 mg/kg) (Table 1).

### 2.6. Acute Antidepressant Activity and Possible Mechanism of Action of BP

**Figure 8:**
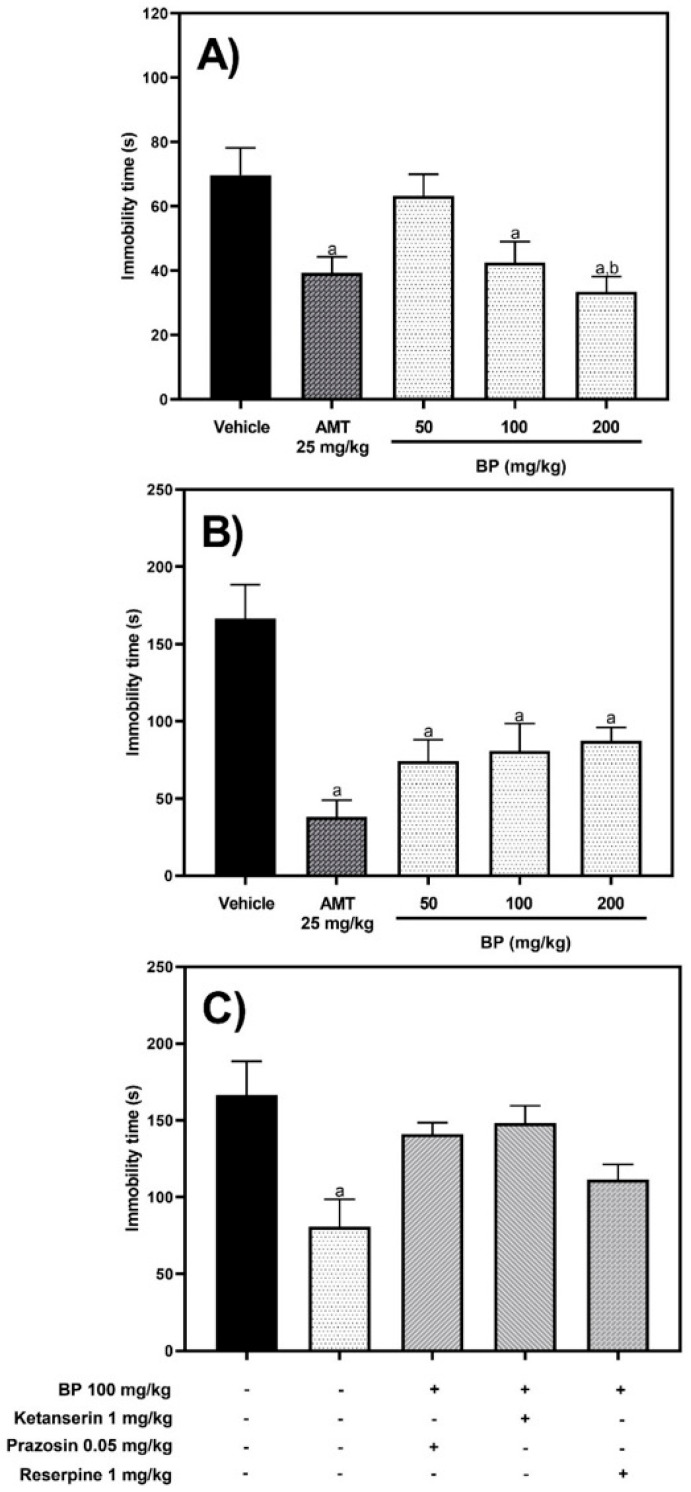
Antidepressant activity of BP (50, 100, and 200 mg/kg p.o.) was evaluated in FST (**A**), TST (**B**), and the mechanism of action of BP with inhibitors in the TST (**C**). The reference drug was 25 mg/kg p.o. amitriptyline (AMT). The results of all experimental groups are reported as the mean values (±SEM) (n = 8). Data were analyzed using a one-way analysis of variance (ANOVA) followed by Tukey post hoc tests. In the FST: ^a^ *p* < 0.05 vs. the vehicle group, and ^b^ *p* < 0.05 vs. the BP group at the dose of 50 mg/kg. In the TST: ^a^ *p* < 0.0001 vs. the vehicle group. In Figure 8C: ^a^ *p* = 0.0090 vs. the vehicle group.

In the forced swimming test (FST), one-way ANOVA analysis exhibited significant differences between experimental groups [F (4,30) = 6.246, *p* = 0.0009]. AMT (25 mg/kg p.o.) and BP (100 and 200 mg/kg p.o.) reduced the immobility time compared to the vehicle group by 43.58% (*p* = 0.0243), 38.9% (*p* = 0.0412), and 53.11% (*p* = 0.0023), respectively (Figure 8A), without showing any difference between these treatments (*p* > 0.05). BP (200 mg/kg p.o.) decreased (*p* < 0.05) immobility time with higher activity compared to BP (50 mg/kg p.o.) (Figure 8A).

In the tail suspension test (TST), one-way ANOVA analysis revealed significant differences between experimental groups [F (4,48) = 9.169, *p* < 0.0001]. AMT (25 mg/kg p.o.) or BP at doses of 50, 100, and 200 mg/kg showed a reduction in immobility time by 77.1%, 55.5%, 51.5%, and 47.51% (*p* < 0.0001 to <0.01), respectively, compared to the vehicle group. However, the antidepressant activity among these groups was the same (*p* > 0.05) (Figure 8B).

On the other hand, TST was used to evaluate the mechanism of action of BP antidepressant activity (Figure 8C). One-way ANOVA analysis exhibited significant differences between the different groups [F (4,38) = 3.500, *p* = 0.0158]. The reduction (51.5%) in immobility time in mice by 100 mg/kg BP (*p* = 0.0090) was reversed by the co-administration of ketanserin (1 mg/kg), prazosin (0.05 mg/kg), and reserpine (1 mg/kg). These treatments showed no difference compared to the vehicle group (*p* > 0.05) (Figure 8C).

**Figure 9:**
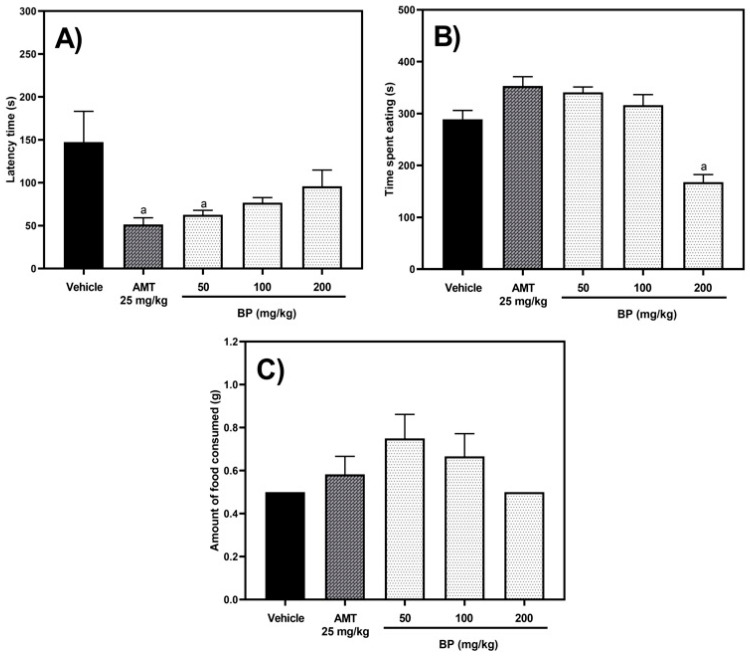
Effect of BP in the Novelty-Suppressed Feeding Test. The parameters evaluated were (**A**) latency time, (**B**) time spent eating, and (**C**) amount of food consumed. The abbreviations of AMT and BP correspond to amitriptyline (positive) and butylidenephthalide, respectively. The results are reported with the mean ± standard error mean (SEM) (n = 6–8). All data were analyzed using a one-way analysis of variance (ANOVA) followed by Dunnett post hoc tests. The “a” letter means statistical difference (*p* < 0.05) versus the vehicle group.

In the Novelty-Suppressed Feeding Test (NSFT), one-way ANOVA analysis revealed significant differences between the experimental groups in the latency time [F (4,25) = 3.964, *p* = 0.0127] and the time spent eating [F (4,24) = 18.29, *p* < 0.0001], but not in the amount of food consumed [F (4,25) = 1.932, *p* = 0.1363]. The experimental groups treated with AMT (25 mg/kg p.o.) and BP (50 mg/kg p.o.) exhibited a reduction in latency time of 65.08% (*p* = 0.0380) and 57.49% (*p* = 0.0430), respectively, when compared to the vehicle group (Figure 9A). Additionally, only BP (200 mg/kg p.o.) led to a 41.94% reduction in the time spent eating compared to the vehicle group (*p* = 0.0026) (Figure 9B). However, the total amount of food consumed by the experimental groups did not differ significantly (*p* > 0.05) (Figure 9C).

### 2.7. Effects of BP on the Chronic Depression Model Induced by Reserpine: Antidepressant Activity and Locomotor Action

**Figure 10:**
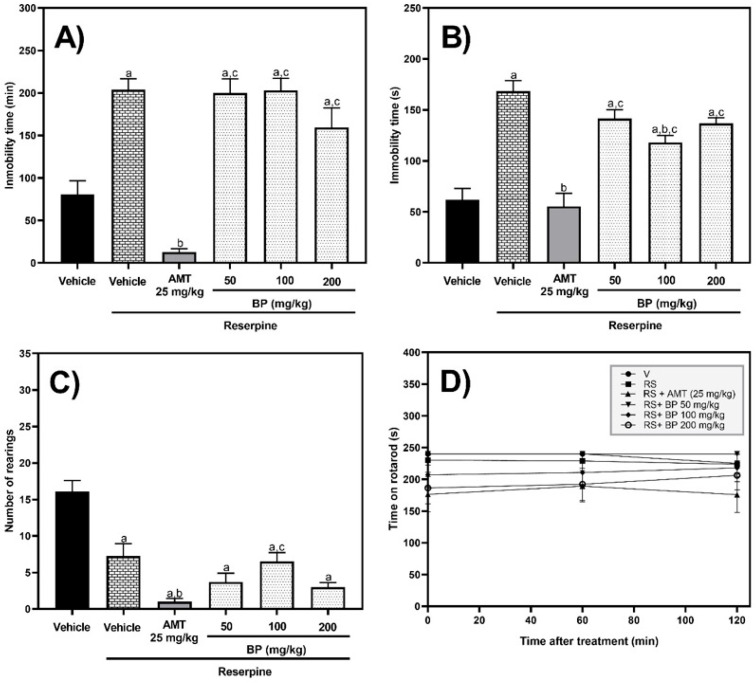
Behavioral tests of the reserpine-induced chronic depression model. The figures (**A**–**D**) correspond to the TST, FST, ECT, and rotarod tests, respectively. The abbreviations V, RS, BP, and AMT correspond to vehicle, reserpine, butylidenephthalide (BP), and amitriptyline, respectively. The results are reported with the mean ± standard error of the mean (SEM) (n = 10). Data were analyzed using a one-way analysis of variance (ANOVA) followed by Tukey post hoc tests. The “a” letter means statistical difference (*p* < 0.05) versus the vehicle group not treated with reserpine, “b” means statistical difference versus the vehicle group pretreated with reserpine (*p* < 0.05), and “c” means statistical difference versus the AMT group (*p* < 0.05).

In the tail suspension test (TST), one-way ANOVA exhibited significant differences between experimental groups [F (5,39) = 32.64, *p* < 0.0001]. The vehicle and BP (50–200 mg/kg p.o.) groups that were pretreated with reserpine exhibited longer (*p* < 0.01) immobility times than the vehicle group that did not receive reserpine. However, treatment with AMT in conjunction with reserpine significantly reduced (*p* < 0.0001) the immobility time by 93.8% compared to the reserpine group. Notably, the maximum percentage reduction in immobility time achieved by 200 mg/kg BP and reserpine was 21.96%, which was not statistically different (*p* > 0.05 vs. the reserpine group) (Figure 10A).

In the forced swimming test (FST), one-way ANOVA also exhibited significant differences between experimental groups [F (5,36) = 21.10, *p* < 0.0001]. The group treated with amitriptyline and reserpine exhibited immobility times that were comparable (*p* > 0.05) to those of the vehicle group that did not receive reserpine. In contrast, the other groups, including the reserpine group and those receiving reserpine with BP at different doses, displayed increased immobility times, ranging from 1.9 to 2.7 times higher than that of the vehicle group without reserpine. Furthermore, only the AMT (25 mg/kg p.o.) and BP (100 mg/kg p.o.) groups significantly reduced the immobility time by 67.1% (*p* < 0.0001) and 30.0% (*p* = 0.0028), respectively, compared to the reserpine group (Figure 10B).

In the exploratory cylinder test (ECT), one-way ANOVA revealed significant differences between experimental groups [F (5,51) = 19.93, *p* < 0.0001]. All experimental groups with reserpine (vehicle, BP, or AMT) exhibited a decrease (*p* < 0.0001) in the number of rearings compared to the vehicle group (without reserpine). Moreover, the group receiving AMT 25 mg/kg with reserpine significantly reduced the number of rearings by 86.25% and 84.6% compared to the reserpine group (*p* = 0.0066) and BP (100 mg/kg p.o.) with reserpine (*p* = 0.0464) groups, respectively (Figure 10C).

In the rotarod test, no significant differences in the time spent on the rotarod apparatus were observed among the different experimental groups at 0 [F (5,54) = 2.106, *p* = 0.0787], 60 [F (5,54) = 1.496, *p* = 0.2064], and 120 min [F (5,54) = 1.278, *p* = 0.2869] post-treatment (Figure 10D).

### 2.8. Docking Study

Experimental evidence indicated the involvement of adrenergic and serotonergic systems. In addition, BP partially reversed depressive-like behavior in a reserpine-induced depression model, in which reserpine is a known VMAT2 inhibitor. Based on these observations, the α1A-adrenergic receptor (α_1A_-AR), the 5-hydroxytryptamine receptor 2A (HTR_2A_), and the vesicular monoamine transporter 2 (VMAT2) were selected as potential molecular targets. Given independent evidence suggesting a regulatory relationship, blind docking was employed solely to explore whether direct molecular interactions between BP and these proposed protein targets are structurally plausible.

**Figure 11:**
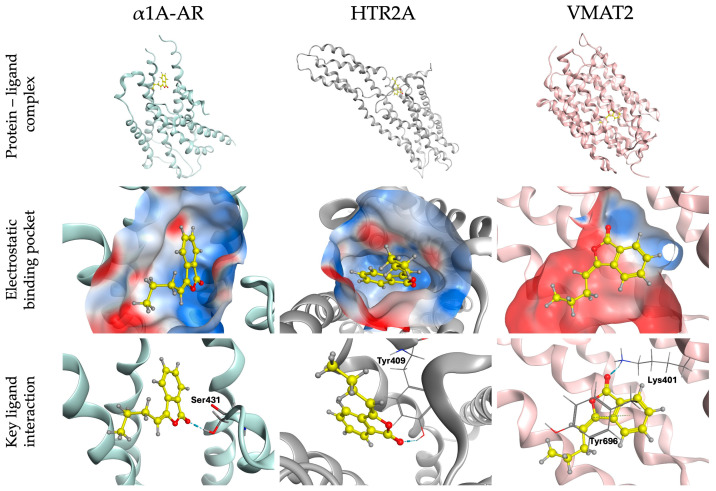
Molecular docking analysis of BP with _α1_A-AR, HTR_2A_, and VMAT2. The upper panels show the overall three-dimensional structures of each protein, with the ligand highlighted in yellow. The middle panels depict BP positioned within the electrostatic binding pockets following docking, represented as semi-transparent molecular surfaces. Electrostatic potential surfaces were calculated using the Poisson–Boltzmann method and visualized using a fixed electrostatic scale to enable direct comparison of the ligand-binding pockets across the three proteins. Red and blue surfaces indicate negative and positive electrostatic potentials, respectively. The lower panels illustrate three-dimensional protein–ligand interaction diagrams, highlighting key amino acid residues in-volved in hydrogen bond interactions (blue dashed lines) and π–π interactions (green dashed lines).

Despite its relatively small molecular size, BP exhibited in silico interactions with _α1_A-AR, HTR_2A_, and VMAT2. Specifically, hydrogen bond interactions were observed with all three proteins, while an additional π–π stacking interaction was identified within the VMAT2 binding site (Figure 11 and Table 2).

Docking scores correspond to GBVI/WSA dG values calculated using MOE. Hydrogen bond distances are reported in angstroms (Å). π–π interactions refer to aromatic stacking between ligand and receptor residues.

Post-docking analysis revealed that the preferred binding poses of BP were consistently located within cavities predicted by Site Finder. These cavities exhibited structural and physicochemical features consistent with relevant binding sites, as detailed in Table 3.

Site size indicates the number of atoms defining the binding pocket. PLB corresponds to the predicted ligand-binding propensity of the pocket, as calculated by MOE. Hyd represents the number of hydrophobic contributions within the binding site, as calculated by MOE. Side chains correspond to the number of atoms from amino acid side chains defining the binding site, excluding backbone atoms.

The docking scores indicate moderate interaction strength, consistent with an exploratory interaction profile rather than high-affinity inhibition. The combined use of global docking and post-docking pocket analysis enabled an unbiased exploration of potential binding regions while supporting ligand localization within structurally favorable cavities predicted by Site Finder.

Electrostatic surface analysis of _α1_A-AR showed that Arg148 (+) and Asp123 (–) flank the binding pocket, generating a locally polarized environment. Consistent with the neutral nature of BP, ligand stabilization is mediated by polar and ion–dipole interactions rather than classical salt bridges, with a hydrogen bond observed between the lactone carbonyl group and Ser431.

In HTR_2A_, residues within 5 Å of BP include Lys65, Lys342, and Glu275, defining a polarized binding pocket. The ligand is accommodated by hydrophobic (Leu66, Leu74, Ala343, Val346) and aromatic residues (Tyr402, Phe405, Tyr409), while polar residues (Asn71, Asn406, Thr408) contribute to specific contacts. A hydrogen bond with Tyr409 further supports ligand stabilization.

In VMAT2, the binding pocket exhibits a polarized electrostatic environment shaped by Lys401 and Glu575. BP is accommodated within a predominantly hydrophobic and aromatic cavity formed by Leu300, Leu491, Ala402, Ala492, Val495, and phenylalanine residues (Phe398, Phe597, and Phe692). Additional polar residues (Asn297, Asn568, Ser601, Tyr604, Cys693, and Tyr696) may further contribute to ligand stabilization. A hydrogen bond with Lys401 and a π–π stacking interaction with Tyr696 were identified, suggesting a binding mode primarily driven by hydrophobic and aromatic interactions, complemented by polar and ion–dipole contributions.

### 2.9. ADMET Analysis

**Figure 12:**
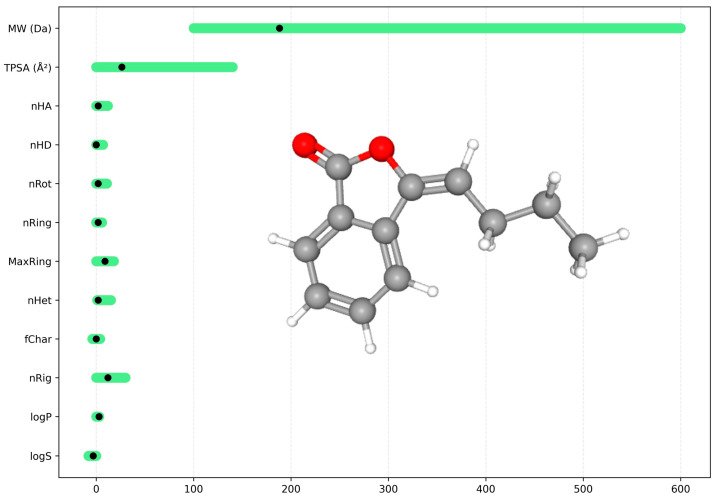
Physicochemical and structural profile of BP showing key drug-likeness parameters, with optimal ranges highlighted in green and the compound values indicated by black markers. The red area in the chemical structure corresponds to oxygen molecules.

The compound exhibits a low molecular weight (MW = 188.08 Da), low polarity (TPSA = 26.3 Å^2^), and moderate lipophilicity (logP = 2.81), fully complying with classical drug-likeness criteria (Lipinski, GSK, and Golden Triangle rules) (Figure 12). These properties suggest good passive membrane permeability and favorable formulation feasibility.

From a chemical accessibility standpoint, the compound shows favorable scores (QED = 0.667; SA score = 2.0). However, its low sp^3^ fraction and limited medicinal chemistry evolution (Fsp^3^ = 0.25; MCE-18 = 20) may restrict pharmacological selectivity and complicate further structural optimization (Table 4).

Structural alert analysis revealed two relevant signals: one ALARM NMR alert associated with thiol-like reactivity and a high probability of chemical reactivity (0.902). Although these features do not disqualify the compound as a drug candidate, they emphasize the need for careful experimental validation to avoid false-positive biological assay results.

Regarding absorption and permeability, BP demonstrates good membrane passage. The Caco-2 and MDCK models predict adequate permeability, whereas the PAMPA model, an artificial membrane assay that evaluates passive transcellular diffusion independently of transporters, indicates low permeability. The compound is not predicted to be a P-glycoprotein (P-gp) substrate, although it is likely a P-gp inhibitor. According to ADMETLab definitions, the Human Intestinal Absorption (HIA) value (HIA + probability = 0.019) corresponds to an intestinal absorption ≥ 30%, suggesting moderate-to-high oral absorption. Additionally, BP shows a high probability of oral bioavailability ≥ 30% and likely ≥50%, indicating acceptable but suboptimal oral bioavailability that could benefit from formulation strategies or structural modifications.

BP exhibited very high plasma protein binding (PPB = 98.8%), resulting in a low free fraction and increased susceptibility to drug–drug interactions due to potential competitive displacement from plasma proteins. A moderate probability of blood–brain barrier penetration is predicted (BBB probability = 0.434), along with a low volume of distribution (VDss), suggesting limited tissue distribution. Furthermore, BP is predicted to inhibit hepatic transporters OATP1B1, OATP1B3, and MRP1, which may have clinically relevant implications, particularly concerning hepatic drug–drug interactions.

From a toxicological perspective, BP shows favorable features, including a low predicted risk of hERG-related cardiotoxicity and low in vitro cytotoxicity in A549, HEK293, and RPMI-8226 cell lines. However, several concerning signals were identified, including moderate-to-high probabilities of drug-induced liver injury (DILI = 0.616), human hepatotoxicity (0.636), induced nephrotoxicity (0.681), and a very high probability of skin sensitization (0.993). A moderate probability of carcinogenicity (0.558) was also predicted.

Tox21 pathways and toxicophore analyses indicate overall low regulatory activity, with no activation of key nuclear receptors such as ER, AR, or PPARγ. Nevertheless, moderate activation of the heat shock element (HSE), associated with cellular stress responses, was observed, along with alerts related to skin sensitization and aquatic toxicity.

## 3. Discussion

Toxicological studies of plant-derived compounds using in vitro and in vivo models provide information about the safety of concentrations/doses used in pharmacological assays. Therefore, the risk/benefit ratio needs to be assessed during drug discovery assays [15]. The acute toxicity test evaluates body weight loss and signs of toxicity, such as piloerection, salivation, and immobility, among others, in rodents. BP showed no effects on the body weight of mice. No macroscopic alterations in organs such as the heart, liver, or kidneys were observed after 15 days of treatment. The LD_50_ value calculated was above 500 mg/kg p.o.

Inflammation is related to the development and progression of mental disorders and neurodegenerative diseases [3]. Preventing inflammation is a target for avoiding cell damage and apoptosis. The formalin and TPA assays are used for the screening of anti-inflammatory agents [16,17]. Topical application of TPA to mouse ears activates protein kinase C and induces neutrophil infiltration, with a subsequent increase in the levels of IL-1β in the epidermis and the release of prostaglandins, leading to the production of edema and epidermal hyperplasia [17]. Furthermore, formalin is a biphasic model consistent across time, where paw licking is the nociceptive response [16]. The first phase (neurogenic) involves the stimulation of nociceptive fibers and the release of histamine, prostaglandins, and bradykinins, whereas the second phase (inflammatory) involves tissue damage and the release of TNF-α, leukocyte migration, and macrophage proliferation in mice paws [16]. The topical application of BP showed a rapid permeation into mouse skin (t_1/2_ = 0.1–1 h), reaching peripheral circulation without accumulating into skin [11]. This work found that the topical application of TPA was not as effective as indomethacin as an anti-inflammatory agent. The oral administration (50–200 mg/kg p.o.) of BP in the TPA and the second phase of the formalin test showed low anti-inflammatory activity compared to indomethacin. Therefore, no further in vivo anti-inflammatory experiments with BP were conducted. The anti-inflammatory effects of BP have been evaluated on in vitro models. After 24 h incubation, BP (20 and 40 µg/mL) decreased the levels of IL-6 and TNF-α, the NF-κB p65 activity, and the levels of p-IKK α/β in LPS-stimulated DC2.4 mouse dendritic cells, which suggests an immunomodulatory effect of BP in inflammatory diseases [18]. After 3 h of TPA administration, the levels of TNF-α increase, promoting vascular permeability [17]. MPO is a marker of neutrophil infiltration of acute inflammation, and its levels increase 6–24 h after TPA exposure [17]. This could be why BP showed no effects on MPO activity. This study provided information that BP decreased the levels of NO, but not MPO activity, in the acute TPA-induced ear edema test. TPA induces the activation of inducible nitric oxide synthetase (iNOS), the enzyme responsible for producing nitric oxide from L-arginine. High levels of NO in mice ears promote edema and vascular permeability with the subsequent activation of NF-κB p65 [19].

This work evaluated the anxiolytic-like, sedative, anticonvulsant, and antidepressant effects of BP to assess the neuropharmacology of this plant compound. Two exploration tests were used to evaluate the anxiolytic-like effects of BP. The hole board test assesses anxiety-like behavior in rodents by observing their tendency to avoid exploring an unfamiliar environment. The head-dip behavior is indicative of anxiolytic-like activity [20]. The cylinder exploratory test consists of simulating a novel environment for mice, which causes rearing and grooming in mice, an indication of anxiogenic behavior. A high number of rearings signals an anxiety-like behavior [13]. BP (50–200 mg/kg p.o.) showed an anxiolytic-like activity that was similar to the activity observed with 1.5 mg/kg CNZ in both the hole board and cylinder exploratory tests.

The pentobarbital-induced sleep test evaluates the hypnotic effects of drugs. The method is based on the drug potentiation in sleep induced by pentobarbital [21]. GABA is released from neurons and inhibits wake-related areas in the hypothalamus. Pentobarbital binds to the GABAA receptor ionophore complex, which opens the chloride channels, inducing membrane hyperpolarization, and causes central nervous system depression [21]. BP (50–200 mg/kg p.o.) did not decrease the latency of falling asleep or prolong the sleep duration, suggesting that this compound lacks hypnotic or sedative effects. The rotarod device evaluates the motor and neuromuscular coordination, muscle synchronization, balance, and depressor activity of the central nervous system in rodents, including effects such as sedation [13]. BP (50–200 mg/kg p.o.) did not affect the balance and motor coordination of mice in the acute assay, suggesting that BP has no effect on neuromuscular coordination.

Pentylenetetrazol is a GABAA receptor antagonist that produces seizures by enhancing neuron activity, suppressing the inhibitory synapse functions, inducing oxidative stress, and increasing cortical malondialdehyde content [22]. BP showed low anticonvulsant effects compared to 1.5 mg/kg CNZ.

The TST and FST are well-established models for evaluating the antidepressant-like activity of drugs [23]. The forced-swimming test allows mice to explore the cylinder filled with water and retrieve olfactory and auditory information, whereas in the TST, the tail is fixed and mice cannot rotate [23]. Depressive-like behavior is estimated by the time that rodents remain immobile. In the acute assays, BP (50–200 mg/kg p.o.) showed antidepressant-like effects comparable to AMT (25 mg/kg p.o.) in both the TST and the FST. Deficiency in norepinephrine and serotonin is associated with a decrease in alertness, pleasure and reward, and low energy, among others, which are symptoms of major depressive disorder [24]. Current pharmacological treatment of major depressive disorders relies on the reuptake inhibitors of norepinephrine and serotonin to increase the levels of neurotransmitters in the brain. These treatments are useful to improve cognitive dysfunction [25]. This work suggests that participation of the serotonergic and adrenergic systems is involved in the antidepressant-like effect of BP in acute assays. The intraperitoneal coadministration of 1 mg/kg ketanserin, a selective inhibitor of HTR_2A_ receptors, or 0.05 mg/kg prazosin, an inhibitor of α_2_ adrenoreceptors, each blocked the antidepressant-like effects of 100 mg/kg BP. These findings indicate the possible participation of the serotonergic and adrenergic systems in the antidepressant-like actions of BP. These results were corroborated when the coadministration of 1 mg/kg reserpine, an inhibitor of vesicular monoamine transporter 2 (VMAT2), which affects the uptake of dopamine, serotonin, and norepinephrine into synaptic vesicles, reversed the antidepressant-like activity shown by 100 mg/kg BP. The in silico assay showed that BP presents favorable affinity for α_1A_-AR, HTR_2A_, and VMAT2. Therefore, the in silico analysis corroborated the in vivo findings, suggesting the involvement of serotonergic and adrenergic systems in the antidepressant-like actions of BP in acute assays. The docking analysis suggests that direct molecular interactions between BP and the proposed protein targets are structurally feasible.

The acute antidepressant-like effects of BP were corroborated with a third behavioral model. A reduction in feeding latency is considered an antidepressant-like activity in NFST [26]. This test evaluates the natural fear of rodents entering an unknown place and exploring it. Food deprivation is an inducer of depressive-like states, simulating anhedonia in humans [27]. BP (50–200 mg/kg p.o.) did not affect appetite in mice, but mice treated with 200 mg/kg BP exhibited less spent time eating compared to all groups, without affecting the amount of food eaten. This study recommended including the time spent eating in this test as a parameter.

Reserpine is an inhibitor of noradrenaline that also depletes the levels of serotonin, dopamine, and catecholamines. Repeated administrations of reserpine in mice induce hippocampal atrophy and a decrease in dendritic spine densities, which are markers for developing depression in humans [28]. In addition, repeated administration of reserpine increases the levels of pro-inflammatory markers such as TNF-α and IL-6, indicating a link between chronic inflammation and depression [28].

Like the acute assay, BP (50–200 mg/kg p.o.) did not affect the locomotor coordination of mice in the reserpine-induced chronic depression model but did not restore the locomotor activity of the vehicle group (mice without reserpine) in the exploratory cylinder test. When BP (50–200 mg/kg p.o.) was tested in multiple applications for several days, the antidepressant-like activity decreased compared to the effects shown by BP in the acute assays; only BP (100 mg/kg p.o.) showed significant antidepressant effects compared to the reserpine group. Due to the low antidepressant-like effects of BP on the reserpine-induced chronic depression model, no further molecular or biochemical assays were performed. Long-term studies evaluated the neuropharmacological effects of BP (50–120 mg/kg p.o.) on models of spinocerebellar ataxia type 3 and dementia [8,10]. However, this work showed that BP decreased its antidepressant-like effects compared to the acute assay when orally administered for 9 days. BP (20 mg/kg p.o.) showed the following pharmacokinetic parameters when administered to rats: t_max_ = 0.22 ± 0.06 and C_max_ = 3 ± 1 µg/mL, indicating that BP plasma levels declined rapidly. The area under the plasma concentration–time curve was 32 ± 1 (µg/mL) * h [29]. There is limited information on the pharmacokinetic profile of BP. Therefore, this work evaluated an ADMET analysis. In terms of excretion and using the in silico approach, BP displayed low-to-moderate clearance (4.9 mL/min/kg) and a short half-life (T_½_ ≈ 1.2 h), indicating that frequent dosing or structural optimization would be required to prolong systemic exposure. Therefore, it may be necessary to conduct additional daily administrations of BP to enhance the antidepressant-like effects in subacute and subchronic studies.

The in silico findings indicate that BP showed a high probability of instability in human liver microsomes (HLM = 0.976), suggesting rapid or unpredictable metabolism and a potential risk of secondary hepatotoxicity. These results corroborate the pharmacokinetic study showing that BP is mainly distributed in the kidney and liver of rats [29]. Metabolism predictions indicate a high probability of inhibition of CYP1A2, CYP2C19, and CYP2C8. CYP2C9 is predicted to act either as an inhibitor or substrate, which is particularly relevant due to the high prevalence of CYP2C9 polymorphisms (*2, *3, among others) [30]. This variability may lead to substantial interindividual differences in systemic exposure (AUC) and increase the risk of interactions with frequently prescribed drugs that rely on or modulate CYP2C9.

The in silico docking and ADMET analyses were conducted as exploratory tools to assess structural feasibility and identify potential pharmacokinetic liabilities, rather than to establish definitive mechanisms of action or therapeutic profiles. Consequently, experimental validation using in vitro models is necessary to corroborate these findings.

## 4. Materials and Methods

### 4.1. Reagents and Drugs

n-Butylidenephthalide (BP) (95% purity) was from Santa Cruz Biotechnology (Santa Cruz, CA, USA); dimethyl sulfoxide (DMSO), TPA (12-O-tetradecanoylphorbol-13-acetate), pentylenetetrazol, pentobarbital, ketanserin, prazosin, and reserpine (RES) were obtained from Sigma-Aldrich (St. Louis, MO, USA); and amitriptyline (AMT) was from Cayman Chemical (Ann Arbor, MI, USA). BP was solubilized in a mixture containing Tween 20 and water [1:9].

### 4.2. Animals

Male and female BALB/c mice (25–35 g) were obtained from the University of Guanajuato’s bioterium and housed in cages (n = 5) at 20 ± 5 °C and a relative humidity of 55% with access to LabDiet 5001^®^ food and water ad libitum in an environment with a 12:12 h light–dark cycle. The University of Guanajuato’s Research Ethics Committee reviewed and approved the experimental protocol with registration number CIBIUG-P60-2024 on 21 October 2024. The experiments were carried out in accordance with the Mexican legislation regarding the technical specifications for the production, care and use of laboratory animals (NOM-062-ZOO-1999). The animals were acclimatized for a period of 2 weeks before starting each experimental protocol. This work reduced, as much as possible, all stress, pain, discomfort, and suffering in mice. The PASS v.6 program (NCSS, Kaysville, UT, USA) calculated the control and experimental sample sizes. A beta value of 0.2, an alpha risk of 0.05, and a two-tailed test type were considered. Female mice were used in the reserpine-induced chronic depression model, whereas male mice were used in the other experimental models.

### 4.3. TPA-Induced Ear Edema

BP was administered topically (0.5–2 mg/ear) or orally (50–200 mg/kg), whereas indomethacin (2 mg/ear or 10 mg/kg p.o.) served as a reference drug. Each group consisted of 8 animals. One hour later, ten microliters of a solution containing 2.5 µg TPA, dissolved in acetone, was applied topically to the inner and outer ear surfaces of each mouse’s ears. After 6 h, the mice were euthanized, and 6 mm diameter sections of right and left ear tissue were obtained and weighed [17]. Ear edema inhibition was obtained using the following equation:

Inhibition (%) = [[Δw control − Δw treatment]/ Δw control] × 100%

where Δw = wt − wnt, wt is the weight of the treated ear section, and wnt is the weight of the non-treated ear section.

#### Estimation of Myeloperoxidase (MPO) Activity and Nitric Oxide (NO) Levels in Ear Homogenates

Tissue homogenization and protein quantification were performed following the protocol described by Barragan-Galvez et al. [31]. The estimation of the MP activity and the NO levels was conducted using the procedures described by Barragan-Galvez et al. [31]. Myeloperoxidase activity and nitric oxide were determined using an ELISA reader with primary filters set at 630 nm and 492 nm, respectively, without using a differentiating filter. The results for MPO activity were expressed as mOD/mg of protein for MPO activity, and the nitrite concentration was determined by extrapolation from the sodium nitrite standard curve (7–100 μM).

### 4.4. Formalin Test

Groups of mice (n = 8 per group) received tramadol (30 mg/kg p.o.), BP (50–200 mg/kg p.o.), or vehicle (saline solution). After one hour post drug administration, twenty microliters of 3% formalin solution (*v*/*v*) were subcutaneously injected into the mice’s right paws. Then, mice were placed into acrylic cylinders. Mirrors were placed next to the acrylic cylinders to observe all movements of the mice’s paws. The time spent licking each mouse’s paws was manually recorded [16].

### 4.5. Pharmacological Treatment in Behavioral Tests

Groups of mice (n = 8 per group) received clonazepam (CNZ, 1.5 mg/kg p.o.), BP (50–200 mg/kg p.o.), or Tween 20 and water [1:9] (vehicle). In the antidepressant-like effects, amitriptyline (AMT, 25 mg/kg p.o.) was used as a reference drug. After one hour of post-drug administration, behavioral tests were performed. BALB/c mice of similar ages were assigned to groups, each with 8 animals. No randomization sequence was used. To reduce the number of animals used in this study, mice underwent experimental models such as pentobarbital-induced sleeping, rotarod, and hole board tests. Two of these behavioral models were performed per week. All behavioral testing and scoring were performed by experimenters who were blind to each pharmacological treatment.

#### 4.5.1. Hole Board Test

A hole board apparatus measuring 50 cm × 50 cm × 20 cm high, containing 16 holes of 2 cm diameter, was used. Each mouse was positioned in the middle of the hole board, and the number of head dips into the holes of each mouse was monitored manually for 5 min. A head dip was considered when each mouse introduced its head completely into a hole [20].

#### 4.5.2. Exploratory Cylinder

Mice were individually placed in open acrylic cylinders (45 cm high, 20 cm in diameter, with a 3 mm wall). The number of hind paw lifts and the number of times each mouse touched the cylinder wall with both forelimbs over a 5-min period were recorded [13].

#### 4.5.3. Pentobarbital-Induced Sleeping Test

A hypnotic dose (40 mg/kg i.p.) of sodium pentobarbital was administered to mice one hour after oral administration of the vehicle or BP (50–200 mg/kg). Then, the mice were placed individually in acrylic cylinders. The onset of sleep was recorded from the time of the intraperitoneal application of pentobarbital until the loss of righting reflexes. The duration of sleep was recorded from the point of loss of the righting reflex until the mice regained it [21]. The recordings were monitored manually.

#### 4.5.4. Rotarod Test

The test utilized a rotating rod apparatus (Panlab Harvard apparatus, Holliston, MA, USA) set to a constant speed of 4 revolutions per min. Mice underwent training for four consecutive days before the test to remain on the rotarod device. On the day of the motor coordination test, the animals were placed on the rotarod, and the latency to fall was recorded with a maximum cut-off time of 240 s, evaluated at 0, 60, and 120 min [13].

#### 4.5.5. Pentylenetetrazol-Induced Seizure

This model evaluates seizures in mice as a screening for compounds with anticonvulsant effects. One hour after drug administration, mice are individually placed in acrylic cylinders for observation. Then, an intraperitoneal administration of pentylenetetrazol (90 mg/kg) induces convulsions represented as involuntary movements in the face, mouth, and front limbs. The latency and duration of seizures and deaths were manually registered for 30 min [22].

#### 4.5.6. Forced Swimming Test (FST)

Animals were placed individually in clear plastic cylinders measuring approximately 25 cm in height by 10 cm in diameter, containing water (temperature approximately 25 °C) at a depth of 15 cm. The test lasted 6 min. The final 4 min were recorded, including the total immobility time for each animal [23].

#### 4.5.7. Tail Suspension Test (TST)

Adhesive tape was placed 1 cm from the tip of the mouse’s tail, and they were suspended 50 cm from the floor on the edge of the table. Immobility time (s) was assessed for 6 min [23]. This test was used to evaluate the potential antidepressant effect, both acute and chronic (in the reserpine-induced chronic depression model on day 17).

Ketanserin (1 mg/kg i.p.), prazosin (0.05 mg/kg i.p.), or reserpine (1 mg/kg i.p.) was used to determine the mechanism of action of BP. Fifteen min later, 100 mg/kg BP was orally administered, and 1 h later, the TST was performed.

#### 4.5.8. Novelty-Suppressed Feeding Test (NSFT)

The protocol of Fukumoto et al. [25] was followed for this test, with some modifications. Mice were restricted from food for 24 h before the test. An acrylic box (42 × 42 × 30 cm), covered with 1 cm of wooden bedding, was used for this test. Three days before the test, mice were habituated to the acrylic box for 30 min. On the day of the test, a small piece of mouse chow (10 cm diameter) was weighed and placed in the center of the box. Each mouse was placed in a corner of the acrylic box under lower lighting (800 lux), and the latency time (measured in seconds) was defined as the duration from when each mouse was released in the acrylic box until it took the first bite of the chow. This study also recorded the time (measured in seconds) spent by the mouse eating the chow and the amount (g) of food consumed. Each session lasted 10 min. Each session was recorded by two experienced observers, and after each test, the cage was cleaned with 70% ethanol to eliminate odor traces.

### 4.6. Reserpine-Induced Chronic Depression in Mice

Female BALB/c mice of similar age were assigned to 6 groups, each with 10 animals. No randomization sequence was used. The RES group consisted of mice that received RES for 10 days (2 mg/kg i.p. for 2 days and 1.5 mg/kg i.p. for 8 days) [32]. Animals treated with RES i.p. received AMT (25 mg/kg p.o.) or BP at 50, 100, and 200 mg/kg orally for 10 days starting on day 11, and the vehicle group consisted of the oral administration of saline solutions (untreated mice). BP, RES, and AMT were solubilized in saline solutions. Behavioral tests were performed on days 17, 18, and 19. After 19 days, the animals were euthanized. This protocol was previously standardized in our laboratory, and all animals developed depression-like symptoms. No mice were excluded from this study. The behavioral tests, including cylinder exploratory, rotarod, FST, and TST, were performed on days 17, 18, and 19, respectively, following the procedures previously described.

### 4.7. Molecular Docking

The three-dimensional structures of the _α1_A-AR, HTR_2A_, and VMAT2 proteins were obtained from the Protein Data Bank (PDB IDs: 7YM8, 6WGT, and 8THR, respectively) [33,34,35]. All peptides and co-crystallized ligands were removed, leaving only the target proteins. Protein preparation and molecular docking analyses were performed using the Molecular Operating Environment (MOE; Chemical Computing Group) software, version 2020.09 (Chemical Computing Group ULC, Montreal, QC, Canada).

Each protein was protonated, assigned partial charges, and subjected to energy minimization using the AMBER10-EHT force field. Docking calculations were performed using the entire protein structure as the receptor, without defining a binding site a priori, allowing for an unbiased exploration of potential ligand-binding regions.

Three-dimensional conformations of BP were generated, protonated, and assigned partial charges. Initial ligand placement was performed using the Alpha Triangle algorithm with the London dG scoring function. Final pose refinement was carried out using the rigid receptor protocol and evaluated with the GBVI/WSA dG scoring function.

Docking poses were subsequently analyzed in relation to binding cavities identified using the Site Finder module in MOE, which were further characterized using structural and physicochemical descriptors.

Protein–ligand complexes were prepared in MOE using QuickPrep and Protonate3D at physiological pH. Water molecules beyond 4.5 Å were removed, followed by restrained energy minimization (RMS gradient 0.1 kcal·mol^−1^·Å^−2^). Electrostatic surface potentials were calculated for the receptor using the Surfaces and Maps module and restricted to residues within 4.5 Å of the ligand, visualized using a red-white-blue color scale based on the Poisson–Boltzmann method. Residues within 5.0 Å of the ligand were identified by distance-based selection and classified according to their protonation states.

### 4.8. ADMET Parameter Analysis of BP

The ADMET parameters of BP were evaluated using ADMETLab 3.0 (https://admetlab3.scbdd.com/server/evaluationCal; accessed 21 December 2025). The SMILES representation of the compound was submitted to generate the corresponding ADMET report.

### 4.9. Statistical Analysis

All data were expressed as the mean ± standard error of the mean (SEM) and analyzed using a one-way analysis of variance followed by Dunnett’s or Tukey’s multiple comparisons test. The F values of one-way ANOVA were calculated to determine the variation between experimental groups. The statistical software used was GraphPad Prism (version 9.5.0). The probability value of *p* < 0.05 was considered a statistical difference.

## 5. Conclusions

BP (50–200 mg/kg p.o.) showed antidepressant-like effects with comparable effects to AMT (20 mg/kg p.o.) in acute assays, with the possible participation of the serotonergic and adrenergic systems. BP lacked hypnotic effects or motor impairment and showed low anticonvulsant and anti-inflammatory effects in the TPA-induced ear edema and the formalin-induced paw edema tests. The antidepressant-like effects shown by BP were lower in the reserpine-induced chronic depression model than in the acute assays.

## Figures and Tables

**Table 1:** Anticonvulsant activity of BP on mice treated with 90 mg/kg pentylenetetrazol to induce convulsions.

Treatment	Onset of Convulsion (s)	Duration of Convulsion (s)	Mortality (%)
90 mg/kg (i.p.) Pentylenetetrazole			
Vehicle	46.81 ± 2.27	119.90 ± 25.44	100.00
CNZ 1.5 mg/kg	216.0 ± 22.08 ***	8.96 ± 3.17 **	0.00
BP 50 mg/kg	57.57 ± 6.02 ^+++^	87.38 ± 27.49	25.00
BP 100 mg/kg	50.14 ± 9.8 ^+++^	75.63 ± 15.41	50.00
BP 200 mg/kg	63.71 ± 5.15 ^+++^	67.38 ± 20.61	50.00

The results of all experimental groups are reported as the mean values (±SEM) (n = 8). CNZ-Clonazepam, BP n-butylenephthalide. *** *p* < 0.0001 and ** *p* = 0.0013 vs. the vehicle group. ^+++^ *p* < 0.0001 vs. CNZ group.

**Table 2:** Summary of protein–ligand interactions between BP and _α1_A-AR, HTR_2A_, and VMAT2 obtained from molecular docking analysis.

Protein	Docking Score (kcal/mol)	Ligand	Receptor Residue	Interaction Type	Distance Å
α_1A_-AR	−5.1553	O (2)	OG (Ser431)	Hydrogen bond H-acceptor	2.67
HTR_2A_	−5.3811	O (2)	OH (Tyr409)	Hydrogen bond H-acceptor	2.76
VMAT2	−6.7296	O (2)	NZ (Lys401)	Hydrogen bond H-acceptor	2.91
		6-ring	6-ring (Tyr696)	π–π	4.15

**Table 3:** Physicochemical and structural characteristics of the BP binding sites in α1A-AR, HTR2A, and VMAT2.

Protein	Size	PLB	Hyd	Side	Residues
_α1_A-AR	66	2.99	20	43	His82, Ser83, Thr85, His86, Asp123, Arg148, Gly151, Val152, Tyr158, Val232, Ala235, Arg368, Lys371, Ala372, Thr375, Leu276, Ser431, Ser432, Gln433, Glu334
HTR_2A_	61	2.77	27	49	Lys65, Leu66, Thr70, Asn71, Leu74, Lys273, Glu275, Asp276, Asn359, Glu340, Lys342, Ala343, Val346, Leu347, Val401, Tyr402, Phe405, Asn406, Thr408, Tyr409
VMAT2	81	3.07	51	78	Asp289, Asn297, Leu300, Phe398, Lys401, Ala402, Gln405, Ser459, Ser463, Leu491, Ala492, Val495, Ile571, Ala572, Glu575, Pro576, Phe597, Ala600, Ser601, Tyr604, Asp689, Phe692, Cys693, Tyr696, Pro700

**Table 4:** Summary of ADMET properties of BP.

Category	Parameter	Predicted Value/Interpretation
Physicochemical	Molecular weight (MW)	188.08 Da
	TPSA	26.3 Å^2^
	logP	2.81
	Drug-likeness	Complies with Lipinski, GSK, Golden Triangle
Chemical accessibility	QED	0.667
	SA score	2.0
	Fsp^3^	0.25
	MCE-18	20
Absorption	Caco-2/MDCK	Adequate permeability
	PAMPA	Low permeability
	P-gp substrate	No
	P-gp inhibitor	Likely
	HIA	≥30% intestinal absorption
	Oral bioavailability	Likely ≥ 30%, probably ≥ 50%
Distribution	PPB	98.8% (very high)
	BBB penetration	Moderate (probability = 0.434)
	VDss	Low
	Transporter inhibition	OATP1B1/OATP1B3/MRP1
Metabolism	CYP inhibition	CYP1A2, CYP2C19, CYP2C8
	CYP2C9	Possible inhibitor or substrate
	HLM stability	Low (HLM = 0.976)
Excretion	Clearance	4.9 mL/min/kg
	Half-life (T½)	~1.2 h
Toxicity (favorable)	hERG	Low risk
	Cytotoxicity	Low (A549, HEK293, RPMI-8226)
Toxicity (concerns)	DILI	0.616
	Human hepatotoxicity	0.636
	Nephrotoxicity	0.681
	Skin sensitization	0.993 (very high)
	Carcinogenicity	0.558
Tox21 pathways	Nuclear receptors	No activation (ER, AR, PPARγ)
	Stress response	Moderate HSE activation

## Data Availability

The original contributions presented in this study are included in the article. Further inquiries can be directed to the corresponding authors.
